# Soft Material-Enabled Electronics for Medicine, Healthcare, and Human-Machine Interfaces

**DOI:** 10.3390/ma13030517

**Published:** 2020-01-22

**Authors:** Robert Herbert, Jae-Woong Jeong, Woon-Hong Yeo

**Affiliations:** 1 George W. Woodruff School of Mechanical Engineering, Institute for Electronics and Nanotechnology, Georgia Institute of Technology, Atlanta, GA 30332, USA; rherbert7@gatech.edu; 2 School of Electrical Engineering, Korea Advanced Institute of Science and Technology (KAIST), Daejeon 34141, Korea; jjeong1@kaist.ac.kr; 3 Wallace H. Coulter Department of Biomedical Engineering, Parker H. Petit Institute for Bioengineering and Biosciences, Neural Engineering Center, Institute for Materials, Institute for Robotics and Intelligent Machines, Georgia Institute of Technology, Atlanta, GA 30332, USA

**Keywords:** soft materials, wearable electronics, implantable electronics, biodegradable, medical devices, diagnostics, health monitoring, human-machine interfaces

## Abstract

Soft material-enabled electronics offer distinct advantages over conventional rigid and bulky devices for numerous wearable and implantable applications. Soft materials allow for seamless integration with skin and tissues due to the enhanced mechanical flexibility and stretchability. Wearable devices with multiple sensors offer continuous, real-time monitoring of biosignals and movements, which can be applied for rehabilitation and diagnostics, among other applications. Soft implantable electronics offer similar functionalities, but with improved compatibility with human tissues. Biodegradable soft implantable electronics are also being developed for transient monitoring, such as in the weeks following surgeries. New composite materials, integration strategies, and fabrication techniques are being developed to further advance soft electronics. This paper reviews recent progresses in these areas towards the development of soft material-enabled electronics for medicine, healthcare, and human-machine interfaces.

## 1. Introduction

Soft material-enabled electronics can address a wide range of applications by enabling a comfortable, continuous, and real-time monitoring of physiological signals via conformal, ergonomic interactions with human tissues when compared to conventional electronics based on bulky and rigid materials. Tissue-friendly, intimate lamination of soft wearable and implantable electronics allow for a long-term, high-fidelity recording of biological signals. An increasing number of materials, integration designs, and fabrication technologies are being developed towards realizing soft electronics due to these advantages. These electronics can enhance healthcare, diagnosis, and therapeutics by offering improved biocompatibility, signal monitoring, and wearability without sacrificing patient comfort. Overall, this special issue collects a number of recent advances in soft material-enabled electronics for medicine, healthcare, and human-machine interfaces.

## 2. Contributions

This special issue has collected thirteen papers with a focus on soft material-enabled electronics for wearable and implantable applications, healthcare, rehabilitation, and more. The main contributions and focused science and technology of each of these papers are outlined in the following.

The overall scope of key materials, enabling technologies, and significant applications of soft material-enabled wearable electronics is detailed in “Soft Material-Enabled, Flexible Hybrid Electronics for Medicine, Healthcare, and Human-Machine Interfaces” [1]. Substrate and sensing material properties are discussed, along with details of the applications for each material. The integration of soft materials with thin films and electronic components to form high performance electronics is included. Discussion regarding the current limitations of wearable electronics provides a view of the future direction of soft electronics.

These wearable electronics utilize a variety of active materials for electrical and sensing functionality. These materials are required to be biocompatible for medical applications. In “Flexible and Stretchable Bio-Integrated Electronics Based on Carbon Nanotube and Graphene” [2], the authors review details of soft electronics while using two biocompatible materials, carbon nanotubes and graphene. Integration strategies of these materials with soft substrates are discussed. A number of applications are detailed, such as neural mapping and human-machine interfaces.

One such example of these wearable electronics is demonstrated in “A Soft Polydimethylsiloxane Liquid Metal Interdigitated Capacitor Sensor and Its Integration in a Flexible Hybrid System for On-Body Respiratory Sensing” [3]. The proposed sensor utilizes a soft material to form channels that are filled with conductive liquid metal. This package forms a soft, interdigitated capacitor that senses both strain and proximity. The soft, conformal nature of the sensor, due to the soft backbone, enables conformal lamination on skin to monitor the breathing signals.

In addition to laminating the soft systems on the skin, wearable electronics can be integrated with clothing. In “Soft-Material-Based Smart Insoles for a Gait Monitoring System” [4], a soft conductive textile is integrated with thin capacitive sensors. The durable, lightweight system is seamlessly integrated into an insole and it provides a comfortable alternative to existing bulky devices. Gait data is wirelessly transmitted for real-time analysis of patients. 

In addition to enabling wearable electronics, soft materials allow for lightweight, portable electronics to enhance healthcare procedures. In “An Optical Biosensing Strategy Based on Selective Light Absorption and Wavelength Filtering from Chromogenic Reaction” [5], a soft material-based biosensor is developed for user-friendly glucose sensing. A soft material biosensing channel uses a chromogenic reaction and it acts as a color-filtering layer. This color filtering layer modifies the image that is captured by a smartphone camera to signal different concentrations of glucose. This portable device is a vast improvement over the conventional high power and complex system.

While soft materials allow for conformal lamination of wearable electronics onto skin, these materials allow for seamless integration of implantable electronics with tissue. In “Advances in Materials for Recent Low-Profile Implantable Bioelectronics” [6], the authors provide a comprehensive review of these implantable electronics and relevant materials. A variety of materials are discussed, which include soft polymers, metals, and biodegradable materials. Challenges and recent advances in fabrication techniques are detailed. A discussion of biodegradable materials for transient electronics is also included.

One type of implantable electronics is focused on in “Biocompatible and Implantable Optical Fibers and Waveguides for Biomedicine” [7]. Implantable optical fibers and waveguides enable the delivery of light into deep tissues. This method allows for biological sensing, stimulation, and therapies, such as optogenetics. An array of materials, including natural materials, such as silk, and fabrication techniques are reviewed in this paper.

Implantable, prosthetic devices have also been developed with soft materials. Such soft materials allow for significantly higher patient comfort. In “Use of Superelastic Nitinol and Highly-Stretchable Latex to Develop a Tongue Prosthetic Assist Device and Facilitate Swallowing for Dysphagia Patients” [8], a soft material-based prosthetic tongue is fabricated for treating dysphagia. Soft materials, integrated with nitinol wires, replicate the elevation function of the tongue to move food to the back of the mouth for proper swallowing. Currently, there are no therapies or devices to replace the swallowing abilities once lost.

Biocompatible materials are studied and developed to continue the advancement of soft implantable electronics. Hydrogels is one such type of material. In “Electroactive Hydrogels Made with Polyvinyl Alcohol/Cellulose Nanocrystals” [9], a hydrogel is enhanced by varying cellulose nanocrystal concentrations. Transparency and electroactive properties are improved, which are promising improvements for biomimetic soft robots and active drug release.

Implantable electronics can also be used for transient applications and eliminating removal surgeries by utilizing biodegradable materials. These transient electronics use soft, biodegradable electronics while matching the mechanical properties of tissues, as discussed in “Materials and Devices for Biodegradable and Soft Biomedical Electronics” [10]. Specific applications include diagnostics, therapeutic, and power supplies, such as energy harvesters. Power supplies are of high interest, as they enable noninvasive powering and communication of data from implanted electronics.

For transient electronics, highly conductive materials are of significant interest in achieving high performance, bioresorbable electronics. In “Processing Techniques for Bioresorbable Nanoparticles in Fabricating Flexible Conductive Interconnects” [11], bioresorbable conductive patterns are screen printed with an optimized paste mixture. A multi-step process is used to achieve highly conductive and flexible patterns for use in transient electronics.

Soft materials can also play a key role for medical procedures, such as robot-assisted surgeries. A soft, sponge tactile sensor is developed in “Material Characterization of Hardening Soft Sponge Featuring MR Fluid and Application of 6-DOF MR Haptic Master for Robot-Assisted Surgery” [12]. This soft sponge is filled with magneto-rheological fluids to replicate the mechanical stiffness of various cells and biological organs. The tactile sensor allows for the surgeon to distinguish different types of sensors while performing robot-assisted surgery. The demonstrations show significant improvements by providing highly accurate feedback to the surgeon.

In addition to the vast number of medical applications, soft material electronics can be extended to other applications. In “Spray Deposition of Ag Nanowire—Graphene Oxide Hybrid Electrodes for Flexible Polymer—Dispersed Liquid Crystal Displays” [13], spray coating is applied to a flexible polymer sheet and then integrated into smart windows. The optical properties of the smart windows are controlled via an electric field to enhance the building efficiency. Existing designs rely on costly and fragile materials as compared to the flexible spray coated material.

In summary, new advancements in soft material-enabled wearable and implantable electronics, covered in this special issue, have significantly improved the device performance, compatibility, and reliability, which allowed for various applications in health monitoring, disease diagnostics, and human-machine interfaces. Continuous study in basic science and technology development in this area will enable smart medicine, home healthcare, and independent living in the near future.
